# Self-sustained rhythmic behavior of *Synechocystis* sp. PCC 6803 under continuous light conditions in the absence of light–dark entrainment

**DOI:** 10.1093/pnasnexus/pgaf120

**Published:** 2025-04-25

**Authors:** Lutz Claus Berwanger, Nikolaus Thumm, Florian Pascal Stirba, Rahil Gholamipoorfard, Alice Pawlowski, Petra Kolkhof, Jeannine Volke, Markus Kollmann, Anika Wiegard, Ilka Maria Axmann

**Affiliations:** Synthetic Microbiology, Heinrich Heine University Düsseldorf, Düsseldorf 40225, Germany; Synthetic Microbiology, Heinrich Heine University Düsseldorf, Düsseldorf 40225, Germany; Synthetic Microbiology, Heinrich Heine University Düsseldorf, Düsseldorf 40225, Germany; Computational Systems Biology of Cancer, University of Cologne, Köln 50931, Germany; Synthetic Microbiology, Heinrich Heine University Düsseldorf, Düsseldorf 40225, Germany; Mathematical Modelling of Biological Systems, Heinrich Heine University Düsseldorf, Düsseldorf 40225, Germany; Plant Biochemistry, Heinrich Heine University Düsseldorf, Düsseldorf 40225, Germany; Mathematical Modelling of Biological Systems, Heinrich Heine University Düsseldorf, Düsseldorf 40225, Germany; Synthetic Microbiology, Heinrich Heine University Düsseldorf, Düsseldorf 40225, Germany; Synthetic Microbiology, Heinrich Heine University Düsseldorf, Düsseldorf 40225, Germany

**Keywords:** cyanobacteria, circadian clock, *Synechocystis* sp. PCC 6803, backscatter, KaiC3

## Abstract

Circadian clocks regulate biological activities, providing organisms with a fitness advantage under diurnal conditions by enabling anticipation and adaptation to recurring external changes. Three proteins, KaiA, KaiB, and KaiC, constitute the circadian clock in the cyanobacterial model *Synechococcus elongatus* PCC 7942. Several techniques established to measure circadian output in *Synechococcus* yielded comparably weak signals in *Synechocystis* sp. PCC 6803, a strain important for biotechnological applications. We applied an approach that does not require genetic modifications to monitor the circadian rhythms in *Synechococcus* and *Synechocystis*. We placed batch cultures in shake flasks on a sensor detecting backscattered light via noninvasive online measurements. Backscattering oscillated with a period of ∼24 h around the average growth. Wavelet and Fourier transformations are applied to determine the period's significance and length. In *Synechocystis*, oscillations fulfilled the circadian criteria of temperature compensation and entrainment by external stimuli. Remarkably, dilution alone synchronized oscillations. Western blotting revealed that the backscatter was ∼6.5 h phase-delayed in comparison to KaiC3 phosphorylation.

**Significance Statement**

Monitoring circadian rhythms in cyanobacteria usually requires genetically modified reporter strains or extensive sampling for downstream analysis. Even in the main cyanobacterial model, *Synechocystis* sp. PCC 6803, the extent to which undamped circadian oscillations are present has been debated, until a suitable reporter strain was developed. We applied online backscatter measurements as an alternative readout to monitor circadian oscillations in cyanobacteria. In *Synechocystis*, the temperature-compensated *kaiA1B1C1*-driven 24 h oscillation did not require light–dark entrainment, highlighting the clock's relevance under continuous light, which is important for industrial set-ups. Our method opens up the possibility of extending circadian analysis to non-genetically modified organisms (non-GMOs) and monitoring rhythmicity during high-density cultivation of different strains.

## Introduction

Natural biological activities follow circadian patterns that allow organisms, from humans to cyanobacteria to adapt to daily environmental changes (1). A circadian oscillator is defined by (i) a self-sustaining oscillation with a period of 24 h, (ii) the ability to synchronize the internal oscillator with external rhythmic stimuli (entrainment), and (iii) a consistent period length over a physiologically relevant temperature range (2, 3). A timing system that enables organisms to predict changes in light before sunrise or sunset, and to adjust the expression of certain genes, such as for photosynthesis, is beneficial (4, 5). Cyanobacteria are well-suited model organisms for studying circadian rhythms and the connection between the clock and metabolism (6–8). Diurnal metabolic and transcriptional changes are associated with the output of the cyanobacterial circadian clock (9, 10). Changes in cellular structures and large particles (e.g. DNA condensation, DNA supercoiling, synthesis, and degradation of glycogen) are regulated by the clock (11–14). In *Synechococcus elongatus* PCC 7942 (*Synechococcus*), the Kai proteins (KaiA, KaiB, and KaiC) form the central oscillator, which regulates gene expression through KaiC phosphorylation and an output apparatus (3, 15–18). The clock of *Synechocystis* sp. PCC 6803 (*Synechocystis*) appears to regulate transcription to a lesser extent (19, 20). Multiple *kai* gene copies exist in *Synechocystis*: *kaiA1*, *kaiA3*, *kaiB1–B3*, and *kaiC1–C3* (21–24). A recent promoter study showed that circadian oscillations diminish after *kaiA1B1C1* deletion (25). KaiA1B1C1 and KaiA3B3C3 form two interconnected systems, and both KaiC1 and KaiC3 display circadian phosphorylation rhythms (23, 25). Glycogen the main storage compound in *Synechocystis* and *Synechococcus* is anabolized during the day and catabolized at night, regulated by clock proteins in *Synechococcus* (14, 26–29). *Synechocystis* displays diurnal patterns of photosynthesis, respiration, gene expression, and glycogen synthesis and degradation (9, 26, 30–34). Coupling of luciferase to a clock-controlled promoter revealed functional circadian clocks in both *Synechococcus* and *Synechocystis*. Bioluminescence rhythms showed a more rapid damping in *Synechocystis* than in *Synechococcus* and displayed only low amplitude in *Synechocystis* (25, 35–39). Kucho et al. (20) demonstrated that <9% of genes oscillated under continuous light (LL) after 12 h dark entrainment. Recently, true circadian and high-amplitude rhythms of promoter activity have been elegantly confirmed using a strong heterogeneous promoter (25). These findings suggest that *Synechocystis* exhibits circadian control, albeit with lower amplitude of endogenous promoters compared to *Synechococcus*, while metabolism and growth might still be under circadian control. Therefore, we established a noninvasive method that can detect circadian oscillations without the need to detect gene expression rhythms.

The growth of liquid bacterial cultures is traditionally approximated from optical density (OD) measurements, in which the detected light transmitted through the bacterial culture correlates with the cell number in the range of 0.1 to 0.3 or 0.5 (40, 41). Above these values, reliable OD measurements require a shorter path length (42) or sampling and dilution of cultures neither of which allows online measurements in culture flasks. Higher cell densities can be monitored online by measuring the amount of light scattered back by the bacterial culture (40, 43). For *Escherichia coli*, backscatter correlates with cell number up to a dry weight of at least 50 g L^−1^ (40, 44). However, backscatter is not only affected by cell numbers but also by cell size, shape, aggregation, and subcellular structures (45). Accordingly, it has been reported that the backscatter-to-cell correlation is specific for strains and growth phases (46). Backscattered light can be detected in shake flasks. The backscattering of light with **λ** = 521 or 600 nm by microorganisms in liquid cultures has been successfully measured (46–49).

Here, we established the detection of circadian oscillations in the photosynthetic organisms *Synechocystis* and *Synechococcus* via online backscatter measurements using custom 730 nm LED sensors. Wavelet and Fourier transformations after processing the backscattered signal demonstrated significance and temperature compensation of the oscillations. Backscatter signal oscillations of *Synechocystis* cultures were absent after *kaiA1B1C1* deletion. KaiC3 phosphorylation rhythms phase-advanced the backscatter by ∼6.5 h, implying regulation of backscatter rhythms by the two Kai oscillators in *Synechocystis*. The rhythms did not require entrainment by environmental cycles; rather, the cells were synchronized by dilution. Our method allows noninvasive live monitoring of non-GMOs in batch cultures under defined conditions. The low glycogen content of the *kaiA1B1C1* mutant and the lack of backscatter oscillations after *glgC* disruption imply dysregulated synchronization in these strains.

## Results

### *Synechocystis* displays 24 h backscatter oscillations in a noninvasive system

**Fig. 1.**
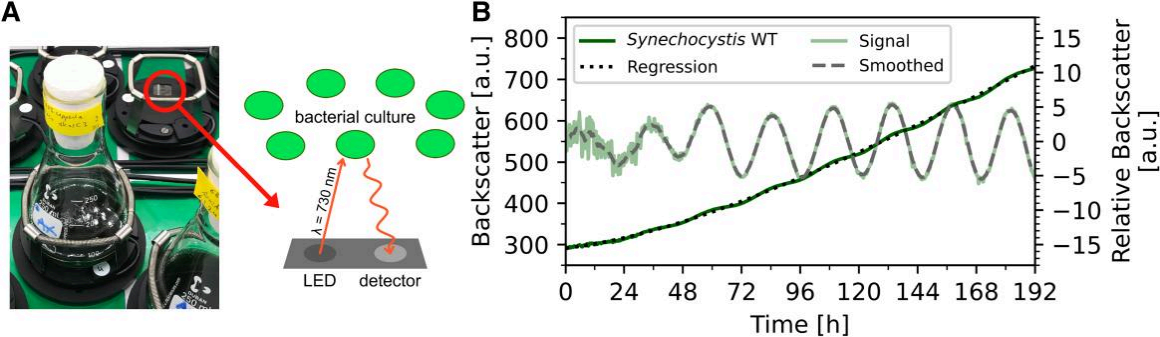
Schematic of the experimental setup and data analysis example. A) Culture flasks were placed on a CGQ sensor platform with an LED emitting 730 nm light into the culture, and a detector (photodiode) detecting any backscattered light. B) Data processing principle for one representative *Synechocystis* WT culture (same as in Figs. 2A and 3A [30 °C]). To eliminate the growth component of the raw backscatter signal (dark green curve, left y-axis), a polynomial regression was fitted to the raw data (dotted curve, left y-axis) and subtracted from the raw data. For normalization, the arithmetic mean was subtracted from the result. The resulting relative backscatter signal was plotted to the right y-axis (light green curve) and the raw signal was smoothed using a moving average to prepare it for further analysis (dashed curve, right y-axis).

For OD measurements of Cyanobacteria, light with a wavelength of 730 or 750 nm is typically used because their pigments do not absorb in this region of the spectrum (42). To monitor the growth of *Synechocystis* and *Synechococcus* using backscatter measurements, we used custom-made cell growth quantifier (CGQ) sensor plates emitting light at 730 nm and detecting any light (using a photodiode) scattered back by a culture placed on the top of the sensor (Fig. 1A).

**Fig. 2.**
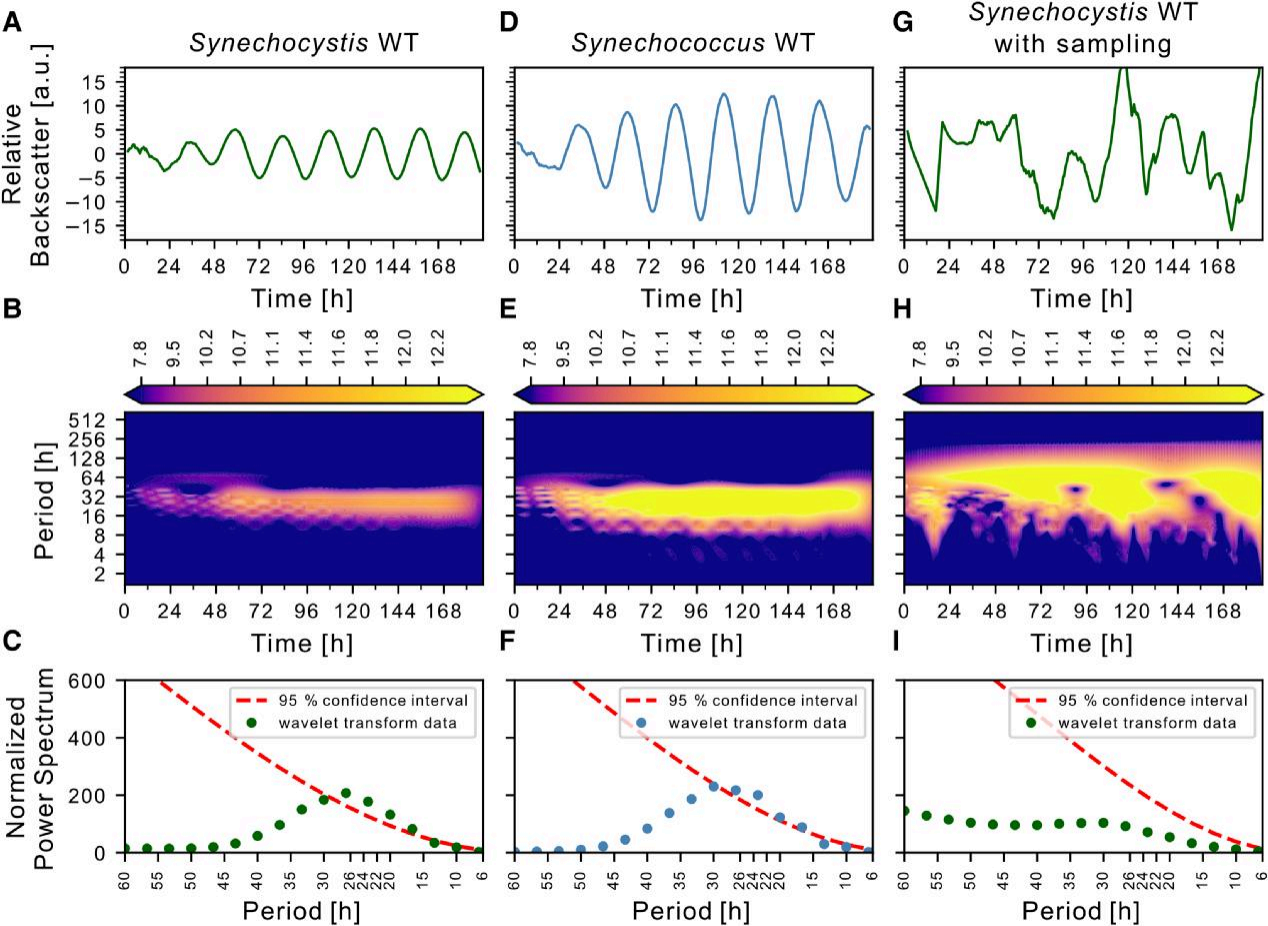
Analysis of *Synechocystis* and *Synechococcus* backscatter signal. A) Representative smoothed backscatter signal of *Synechocystis* WT batch cultures plotted from the initial dilution of the culture (same as Fig. 1B). B) Heatmap of periods over time calculated by wavelet transformation of the extracted signal, with color-scaled coefficient levels. C) Null-hypothesis test for the periods obtained by wavelet transformation (dots) plotting periods (x-axis) against the power spectrum from wavelet transformation (y-axis). Gaussian noise-based curve displayed as “95% confidence interval” (dashed line). D–F) Representative smoothed backscatter signal and downstream processing of *Synechococcus* WT. G–I) Representative backscatter signal and processing after invasive sampling of *Synechocystis* WT batch culture. At 4 timepoints the culture was taken out of the light incubator and returned after taking out 100 μL culture under sterile conditions.

We grew *Synechocystis* WT as two subsequent precultures and then started the experiment by diluting the culture from OD_750nm_ of ∼2 to OD_750nm_ of 0.8 and placing the culture flask on CQG sensors (Fig. 1A) to monitor the backscatter. The backscatter increased over days and despite cultivation under constant conditions, we observed daily changes in raw backscatter signals (dark green curve, Fig. 1B). We removed drift from the measured data on time scales sufficiently longer than the expected 24-h period by fitting a polynomial regression model to the time series and then calculating the difference between the observed and predicted values. Subsequently, the time series was smoothed using a moving average revealing oscillations with a period of approximately 24 h (Figs. 1B and 2A). The backscatter displayed some noise at the beginning of the experiment (light-green curve in Fig. 1B). This phenomenon was previously described for backscatter measurements using shake flasks, and is due to surface reflections, which play a larger role in less dense cultures (47).

Given the detected period length of ∼24, we hypothesized a circadian nature. Repeating the experiment for *Synechococcus*, the main cyanobacterial circadian model organism, also resulted in ∼24 h oscillations (Fig. 2D). The backscatter amplitude of *Synechocystis* WT (∼−5 to 5 a.u., Fig. 2A) was one-third of that of *Synechococcus* WT (∼−12.5 to 12.5 a.u., Fig. 2D). Wavelet transformation (50, 51) revealed recurrent signal oscillations throughout the experimental duration, clustering at periods of 10–45 h for both WT strains (Fig. 2B and E). Comparing these oscillations with the Gaussian-distributed red noise spectrum indicated periods around 24 h were above the 95% CI (Fig. 2C and F). To rule out technical oscillations in the measurement setup, we removed the culture flasks from the sensor and placed it back after taking out a 100 µL sample (Fig. 2G). Taking only four samples over 8 days disturbed the rhythms to the extent that no significant period could be determined anymore (Fig. 2H and I). This experiment also illustrates that sampling needs to be planned with utmost care.

### Oscillations of the backscatter signal are temperature compensated

**Fig. 3.**
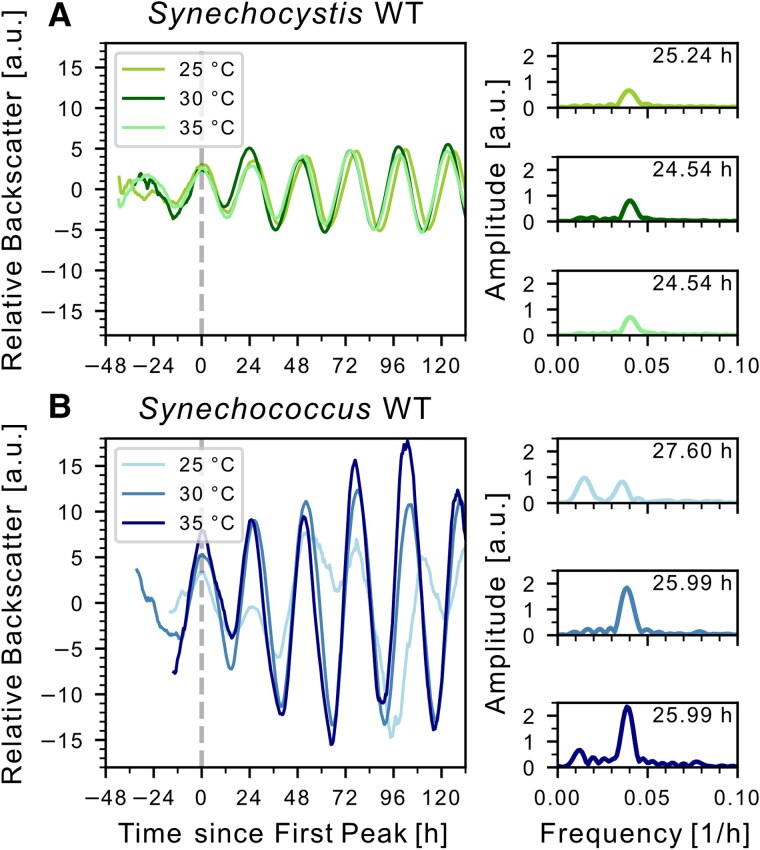
Temperature compensation of backscatter oscillations. A) Left: Representative backscatter signals for *Synechocystis* WT at indicated temperatures. Right: Corresponding frequency spectra of the oscillation signals after Fourier transformation. The highest peaks frequency was used to calculate the period by taking the reciprocal. Q_10_ values were calculated from temperature differences: Q_10 25/30_: 0.95, Q_10_  _30/35_: 1.00. Data shown for 30 °C are the same as in Figs. 1B and 2A. B) Graphs for *Synechococcus* WT. Data for 30 °C are the same as in Fig. 2D. For period determination at 25 °C, the second-highest peak was used, because it was within the range of expectation of a circadian oscillation. Q_10_  _25/30_: 0.89, Q_10_  _30/35_: 1.00. For both strains, the experiment at 35 °C was performed only once.

To investigate whether the ∼24 h oscillations are temperature compensated, we performed backscatter measurements at different temperatures and determined the period for each temperature using Fourier transformation (Fig. 3). For *Synechocystis*, periods did not differ between 25, 30, and 35 °C (Fig. 3A) and displayed a *Q*_10_ value of 0.95–1.00; which is close to previously published values for *Synechocystis* (*Q*_10_ = 1.08–1.1 (25, 34, 35)) and characteristic of circadian clocks (52). Backscatter oscillations of *Synechococcus* WT at 25 °C displayed a reduced amplitude compared to 30 and 35 °C, and the detected periods were not significant according to the wavelet transformation (Fig. 3B). The *Q*_10_ value derived from periods determined at 30 and 35 °C (*Q*_10_ = 1.00) indicated temperature compensation for *Synechococcus* as well.

### Backscatter oscillations can be entrained

**Fig. 4.**
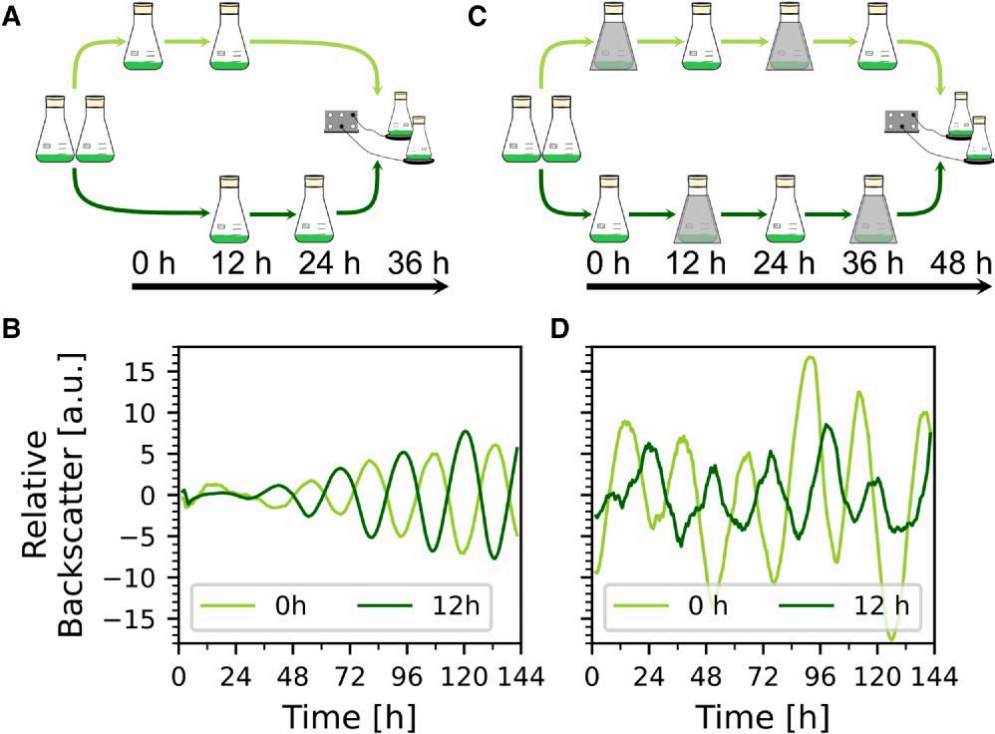
Entrainment of *Synechocystis* WT oscillations. A) Schematic of entrainment by 12 h shifted dilution. B) Representative backscatter signals for *Synechocystis* WT. The first culture was diluted at the time point “0 h” (light green), and the second culture diluted 12 h later (“12 h” dark green). C) Schematic of 12:12 h LD entrainment over 48 h. D) Representative backscatter signals for *Synechocystis* WT. The first culture (light green) was exposed to 12:12 dark:light cycles, over 48 h and the second culture (dark green) with two 12:12 light:dark cycles. 0 and 12 h in the legend indicate the first dark phase postdilution.

Oscillations were observed under continuous light without entrainment by light–dark cycles. We hypothesized that the initial culture dilution served as a nutritional upshift and synchronized cultures. To test this, we diluted one culture with OD_750nm_ ∼ 2 with fresh BG-11 medium (0 h, light green in Fig. 4A) to OD_750nm_ 0.8, and another culture 12 h later from a second preculture (12 h, dark green in Fig. 4A). The cultures were then transferred to the sensor plates. After processing and overlaying the backscatter data of the two cultures, we observed a shift of ∼12 h between the peaks of their backscatter oscillations, indicating successful synchronization (Fig. 4B).

An important criterion for circadian oscillation is that intrinsic rhythm can be synchronized with environmental rhythm. Therefore, we diluted two cultures simultaneously (from OD_750 nm_ of 2 to 0.8) and additionally exposed them to a 12:12 light–dark cycle. One culture received the dark phase directly after dilution (0 h, light green in Fig. 4C), and one culture was exposed to 12 h light first (12 h, dark green in Fig. 4C). If the dark phase was given in phase with the nutritional upshift, stable high-amplitude oscillations were measured (0 h, light green in Fig. 4D). Conversely, if the dark phase was shifted by 12 h, the phase of the backscatter oscillations shifted accordingly (12 h, dark green in Fig. 4D), but oscillation displayed decreased amplitude and was less smooth compared to the culture receiving the dark treatment at the time of dilution, indicating that giving two synchronizing signals simultaneously leads to a stronger amplitude of the rhythms.

### Backscatter oscillations and glycogen synthesis are diminished in a *Synechocystis* clock mutant

**Fig. 5.**
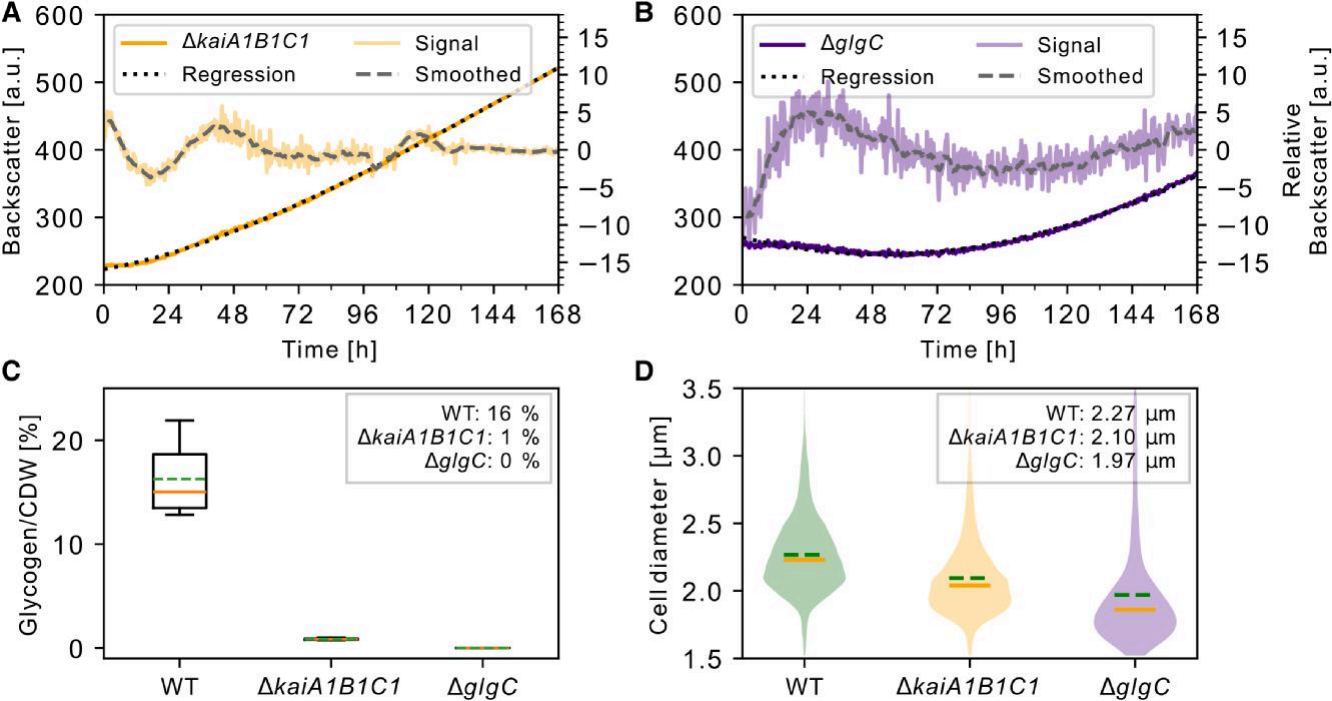
Backscatter and physiological analysis of *Synechocystis* Δ*kaiA1B1C1* and Δ*glgC*. A) Raw backscatter signal of *Synechocystis* Δ*kaiA1B1C1* (orange, left y-axis) with regression (dotted line) and relative backscatter before denoising (signal, light orange, right y-axis) and after smoothing (dashed gray line). B) Backscatter analysis of *Synechocystis* Δ*glgC*: C) Glycogen content per CDW compared to the WT with mean (dashed green lines and legend), median (solid orange lines). Only values above 0 are shown. D) Cell size distribution with mean (dashed green lines, legend), median (solid orange lines) for the indicated strains.

Given that the observed oscillations fulfilled the criteria for circadian oscillations, we sought to test the impact of clock manipulation. As shown in Fig. 5A, deletion of the *kaiA1B1C1* core clock disrupted the oscillation pattern in *Synechocystis*. Carbon metabolism is relevant for circadian synchronization and a glycogen-deficient mutant is more sensitive to environmental changes (14). We created a mutant (*Synechocystis* Δ*glgC*) that was unable to synthesize glycogen by disrupting the *glgC* gene, essential for glycogen biosynthesis ((53) and Fig. 5B). This strain did not display any backscatter oscillations (Fig. 5B) and a prolonged lag phase after synchronization. To estimate the growth rate, we fitted a regression to the backscatter data displayed in Fig. 5B, starting at 108 h. The growth rates of the *ΔglgC* (slope = 1.85 a.u. h^−1^) and *ΔkaiA1B1C1* strains (slope = 2.2 a.u. h^−1^) were lower than those of WT (slope = 2.98 a.u. h^−1^). We measured the glycogen content of different strains from hours 86 to 96 after the nutrition upshift (Fig. 5C). *Synechocystis* Δ*glgC* was unable to synthesize glycogen. *Synechocystis* Δ*kaiA1B1C1* displayed significantly decreased glycogen content compared to WT, suggesting the loss in backscatter oscillations might originate from dysregulated glycogen metabolism and the missing feedback for circadian synchronization. Cell size has been reported to be correlated with glycogen content (54). Accordingly, both mutants, Δ*kaiA1B1C1* and Δ*glgC*, displayed smaller cell sizes than WT (Fig. 5D). Together, these results indicate that the circadian program controlling glycogen synthesis and degradation is malfunctional in the *ΔkaiA1B1C1* mutant, as reflected by the absence of backscatter oscillation.

### KaiC3 phosphorylation phase-advances the backscatter oscillations

**Fig. 6.**
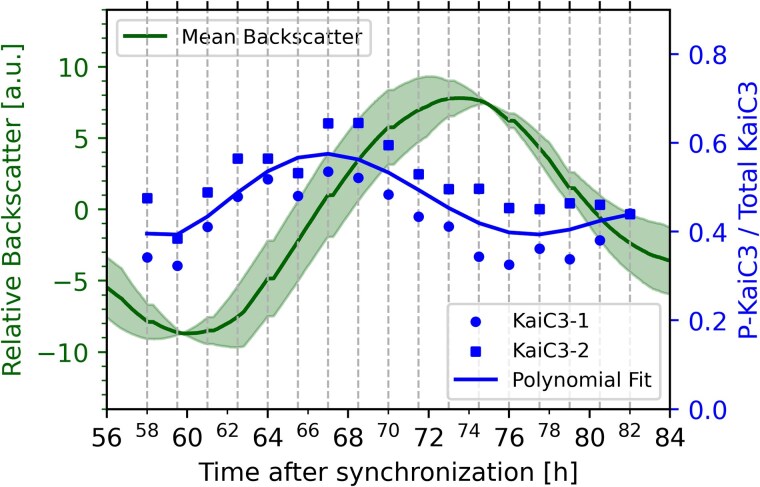
Comparison of backscatter and KaiC3 phosphorylation over 24 h in *Synechocystis*. Fifty-eight hours after dilution, samples for Phos-tag SDS-PAGE followed by western blot analysis, were taken every 1.5 h from individual flasks (dashed vertical lines). Shown is the mean relative backscatter of two biological replicates (dark green, left y-axis), with the shaded area (light green) indicating the range between the replicates for the 28 h surrounding the experiment. These two replicates were not sampled. The ratio of phosphorylated KaiC3 to the total amount of KaiC3 for two technical replicates is plotted to the right y-axis. The blue curve represents a 5th degree polynomial fit to the mean of the two technical replicates (squares and circles) at each time point.

In *Synechocystis*, KaiC1 and KaiC3 display ∼24 h phosphorylation rhythms after entrainment with LD cycles (23), with KaiC3 showing higher amplitude than KaiC1 (23). To test whether these rhythms were also present in our setup, we sampled cells over 24 h. To monitor undisturbed backscatter oscillations, each sample was collected from a separate flask. Whole-cell extracts were separated using Phos-tag SDS-PAGE and subjected to western blotting. Using a KaiC3-specific antibody (22, 23), we detected 24-h oscillations of KaiC3 phosphorylation (Fig. 6), advancing the backscatter rhythms by ∼6.5 h. Our data demonstrate that the backscatter properties of *Synechocystis* cultures serve as a readout of circadian oscillations and can substitute for labor-intensive sampling and processing such as phosphorylation analysis.

## Discussion and outlook

The attractiveness of cyanobacteria as hosts for bioproduction (55, 56) and evidence of oscillations in other prokaryotes (57–59) call for extending the understanding of circadian clocks to other organisms. Most cyanobacterial circadian research relies on live monitoring of reporter strains or labor- and cost-intensive transcriptomic analyses, which may not necessarily overlap with protein or metabolic oscillations (60). Our live monitoring of cyanobacterial growth behavior provides a fast alternative for screening cyanobacteria for circadian rhythms. Backscatter measurements facilitated the monitoring of oscillations in *Synechococcus* and *Synechocystis* over several days without disturbing the culture. Oscillations in the two strains at 30 °C differed by ∼1 h in the exact same setup (Fig. 2A and D), excluding that the periodic fluctuations are due to external cues (e.g. opening of the light incubator). Consistent with a recent study reporting the dependence of promoter activity rhythms on *kaiA1B1C1*, backscatter oscillations were driven by the core oscillator (25). Using the approach established here, we confirmed the dependence of backscatter oscillation on *kaiA1B1C1* using measurements in a microplate bioreactor (23). Very recently, it was also shown for *Cyanothece* that OD displays circadian oscillations, which are dampened in a *kaiA* deletion strain (61).

A light–dark regime or temperature rhythms are typically applied before investigating free-running oscillations (62). We replicated this classic entrainment by two 12:12 h LD cycles, which have been shown to work in *Synechococcus* (35) and *Synechocystis* (34). In addition, our results suggest that dilution synchronizes oscillations in batch cultures. Different effects of external stimuli on entrainment of the circadian clock among different species (e.g. *Neurospora crassa*) have been observed previously (63). Food uptake serves as an entrainment signal (64–66) and, for example influences rhythms of glycogen metabolism in the livers of rats (67) and humans (68, 69). Similarly, an engineered *Synechococcus* strain can be synchronized using rhythmic glucose supply (64). Twenty-four-hour rhythms without LD entrainment have been observed not only in plants and microalgae (including cyanobacteria), in bioreactor-setups, but also in nonphotosynthetic prokaryotes (54, 61, 70, 71).

In *Synechocystis*, the interaction between the KaiA1B1C1 clock system and the output orthologs SasA and RpaA interferes with glycogen metabolism (7, 19, 72, 73) by influencing the switch from photoautotrophy to internal carbon reserve utilization (74). Deletion of *kaiA1B1C1* in *Synechocystis* reduced glycogen content and led to smaller cell sizes compared to the WT, indicating impaired glycogen metabolism. The lack of glycogen storage in *Synechocystis* Δ*kaiA1B1C1* may impair light/dark transitions, as the main role of the circadian clock is to adjust the cell's physiological state to the upcoming night environment (20).

We recently demonstrated that cyanobacterial circadian clocks can be complex, with *Synechocystis* displaying two KaiABC systems driving circadian oscillations together (23). Therefore, measuring a downstream output rather than the core oscillator might be more feasible for organisms, communities, and symbioses with complex or even unknown clock systems (75). Our noninvasive backscatter measurement method is ideally suited for these investigations.

In this study, we detected true circadian rhythms with a free-running period of ∼24 h in *Synechococcus* and *Synechocystis*, linked to the cellular circadian program. The synchronization of oscillations by the initial culture dilution suggests their presence in standard batch cultures. Live monitoring of cyanobacteria holds immense potential for biotechnological applications. Diverse strategies have been developed to manipulate carbon flow in cyanobacteria, and glycogen metabolism has been recognized as a promising target (76–78). This monitoring method might allow researchers to identify optimal time frames for inducing heterologous protein production and synthesizing valuable products by correlating glycogen content, as recently demonstrated for the circadian clock (79). It also facilitates the optimization of growth conditions for cyanobacteria and provides opportunities for the development of innovative biotechnological processes.

## Materials and methods

### Strains construction

To generate the *glgC* (*slr1176*) knockin mutant, a fragment comprising the *slr1176* open reading frame plus 456 nt upstream and 843 nt downstream was amplified using primers JS69 (5′ GGCATCAACGGCGTTGGAAA 3′) and JS70 (5′ GGCACCACTTCCACCGACTG 3′) and ligated into the cloning vector pJET1.2 (ThermoFisher). The kanamycin resistance cassette from pUC-4 K, digested with HincII, was subsequently inserted into the unique PsiI site of *glgC*. Transformation of Glc-tolerant *Synechocystis* sp. strain PCC 6803 (obtained from Marion Eisenhut [University Bielefeld], originally from Prof. N. Murata [National Institute for Basic Biology, Okazaki, Japan]), selection on kanamycin, segregation, and verification via PCR were performed as described in Eisenhut et al. (80).

All analyses were performed in the *Synechocystis* Uppsala (nonmotile strain, derived from Pia Lindberg, Uppsala University) strain background. Mutants were generated by transforming it with gDNA isolated from *Synechocystis* sp. PCC “Murata” *ΔglgC* mutant, and *Synechocystis* sp. PCC-M Δ*kaiA1B1C1* mutant (81). Ten milliliters *Synechocystis* Uppsala (OD_750nm_ of 0.5–1) were pelleted at 2,000 × *g* for 10 min at RT, resuspended in a small amount of supernatant and incubated with 20–50 µg mL^−1^ isolated DNA for at least 30 min at 30 °C before transferring to a BG11 agar plate without antibiotics and incubation for at least 2 h at RT. Cells were grown for 2–3 days, before applying 400 µL of kanamycin (1 mg mL^−1^) to one side of the plate to form a gradient. For segregation, the kanamycin concentration was increased from 25 µg mL^−1^ to 50 µg mL^−1^.

### Backscatter measurements

*Synechocystis* sp. PCC 6803 and *Synechococcus elongatus* PCC 7942 were grown in BG11 (*Protocols.io, dx.doi.org/10.17504/protocols.io.bzjup4nw*) at 0.5% CO_2_, 75% humidity, 150 rpm, with constant light of 80 µmol_photons_ m^−2^ s^−1^ in a Multitron Infors HT^Ⓡ^ incubator at 30 °C, if not indicated otherwise. *Synechocystis* Δ*kaiA1B1C1* and *Synechocystis* Δ*glgC* were supplemented with kanamycin (25 µg mL^−1^).

Backscatter measurements were performed using the CGQ (Aquila Biolabs, now Scientific Bioprocessing [sbi]) system, which consists of one base station, eight sensor plates comprising a flask holder with a photodiode, and live monitoring software. Details of the setup and data processing are described in Bruder et al. (46). To allow the measurement of phototrophic organisms, custom-made sensors with LEDs emitting light of 730 nm wavelength were used (Fig. 1). For each experiment, precultures were initiated from BG11 agar plates and propagated in 20 or 100 mL BG11 in 100 or 250 mL Erlenmeyer flasks, respectively, for at least 5 days. These cultures were diluted to an OD_750nm_ of ∼0.5–1 (Specord 200 Plus, Analytic Jena^©^) and further grown. When the desired OD_750nm_ of ∼2–3 was reached, the cultures were split and diluted to an OD_750nm_ of 0.8 (Figs. 1–5) or 1 (Fig. 6) and placed on the CGQ (sbi) in a Multitron HT Infors incubator. Backscattered light was monitored every 2 min using the CGQuant software (Figs. 1–5) or every 20 s using Dots version 1.3.3 (Fig. 6).

### Cell analysis

For the experiment in Fig. 5, one batch culture (700 mL, OD_750nm_ of 0.8) of *Synechocystis* WT was divided into seven 100 mL cultures, while *Synechocystis* Δ*kaiA1B1C1* and *Synechocystis* Δ*glgC* were each divided into four 100 mL cultures. For the WT, one culture was removed every 2 h over 12 h, starting at 86 h postinoculation. For the Δ*kaiA1B1C1* and Δ*glgC* cultures, one culture was removed every 4 h within the same 12-h period.

To determine the glycogen content, 5 mL of cells were centrifuged in a precooled (−20 °C) reaction tube at 20,000 × *g* for 5 min at 4 °C. After immediately discarding the supernatant, the cell pellets were flash-frozen in liquid nitrogen and stored at −80 °C. For glycogen extraction, following a modified Ernst method (82), pellets were resuspended in 4 mL KOH (30% w/v) and incubated at 95 °C for 2 h. 3 × 400 µL were mixed with 1.2 mL of ice-cold ethanol, and incubated overnight at −20 °C. After centrifugation at 4 °C for 10 min at 10,000 × *g*, the pellet was washed with 70% ethanol and 100% ethanol, dried using a Concentrator Plus Speed-Vac (Eppendorf) for 20 min at 60 °C, resuspended in 1 mL 100 mM sodium acetate (pH 4.5) supplemented with amyloglucosidase powder (Sigma-Aldrich, 10115) to 35 U mL^−1^ and incubated at 60 °C for 2 h. For the spectrometric glycogen determination, the Sucrose/D-Glucose Assay Kit from Megazyme (K-SUCGL) was used according to the manufacturer's specifications, but omitting the fructosidase reaction step and reducing the total reaction volume to 850 µL according to Behle et al. (54). Absorbance was measured at 510 nm using a BMG Clariostar Photospectrometer.

To determine the cell dry weight (CDW), 5 mL of *Synechocystis* culture was sampled on a preweighed Petri dish, dried at 60 °C for 24 h, and weighed after cooling. A Petri dish containing 5 mL dried growth medium (BG11) served as a reference.

To determine cell size, 10 µL of each sample was diluted 1:10 and mixed with 10 mL CASYton (OLS) to a final dilution of 1:10^4^. Measurements were taken in 200 µL in triplicates using a CASY Cell Counter model TTC (Schaerfe Systems), and a capillary with 45 µM aperture size. Cell sizes within a 0–30 µm diameter range were recorded, but only counts with 1.5–5 µm diameter were considered (54). Six replicates for *Synechocystis* WT, four replicates for Δ*kaiA1B1C1*, but only two experiments for the Δ*glgc* strain were conducted, as the capillary frequently clogged during these experiments.

### Detection of in vivo oscillations via phos-tag SDS-PAGE and immunodetection

Cells were synchronized for backscatter measurements. Every 1.5 h 2 mL cell culture from a new flask was centrifuged at 20,000 × *g* for 1 min at 4 °C, flash-frozen in liquid nitrogen and stored at −80 °C. To prevent disruption of backscatter measurements by opening the incubator and stopping the shaking during flask removal, backscatter measurements in the incubator were paused for around 15 min. Cultures were placed in two incubators, each opened only once every 3 h. The backscatter of an additional flask in each incubator, not used for sampling, was monitored. Total protein was extracted as previously described (23). Briefly, cells in lysis buffer (8 M urea, 20 mM 4-(2-hydroxyethyl)piperazine-1-ethanesulfonic acid pH 8.0), were broken by 30 s vortexing and 1 min cooling on ice for seven cycles with ∼200 µL glass beads (0.1 mm, acid-washed), followed by centrifugation at 1,000 × *g* for 3 min at 4 °C. Chlorophyll a content of cell extracts was measured by OD_665nm_ (Implen, Nanophotometer C40) after 30 min extraction in 90% methanol and centrifugation at 20,000 × *g* for 5 min, using the equation: C_chlorophyll_ [µg mL^−1^] = OD_665nm_×12.6 µg mL^−1^ × dilution factor. Cell extracts corresponding to 0.05 µg chlorophyll a were loaded onto a 50 µM L^−1^ SuperSep Phos-tag gel (7.5%, 17 well, Fujifilm Wako Pure Chemical Corporation) and separated at 150 V and 8 °C for 2 h in 25 mM Tris, 192 mM glycine, 0.1% SDS. The gel was washed with water (2–3×), blotting transfer buffer (Trans-Blot turbo transfer buffer, Bio-Rad) supplemented with 10 mM EDTA (2 × 10 min at RT), and blotting transfer buffer without EDTA (1 × 10 min at RT). Proteins were transferred onto a nitrocellulose membrane for 7 min at RT (Trans-Blot Turbo Transfer System, Bio-Rad Laboratories, protocol Mixed MW, 1.3A-25V-7 M). The membrane was blocked with 5% w/v nonfat dry milk/Tris-buffered saline containing 0.1% Tween-20 (TBS-T) for 2 h at RT. Immunodetection used an anti-KaiC3 antibody (1:7500 dilution in TBS-T, 4 °C overnight) (23) and goat anti-rabbit IgG (H + L) Secondary Antibody, HRP (Thermo Fisher Scientific, 1:50,000, 1 h RT). After each antibody incubation, the membrane was washed three times for 10 min in TBS-T. Chemiluminescence detection was performed using Pierce SuperSignal West Pico detection reagent (Thermo Scientific) and ChemiDoc MP Imaging System (Bio-Rad). The ratio of P-KaiC to total KaiC protein was estimated by densitometric analysis of the blot images (Image Lab 6.1, Bio-Rad) and plotted in python.

### Backscatter signal analysis

Data analyses were performed using Python 3.12.3, utilizing numpy (1.26.4), pandas (2.2.3), scikit-learn (sklearn; 1.5.2), scipy (1.14.1), statsmodels (0.14.4), and pywavelets (pywt; 1.7.0). Data visualization was performed using Matplotlib (3.9.1). The growth component of the raw backscatter was removed by fitting a polynomial regression (4th degree) with sklearn.LinearRegression and sklearn.predict functions. This regression was subtracted from the original measurements, and the resulting raw oscillation signal was normalized by subtracting the arithmetic mean. To remove noise from the raw signal, a moving average (window size: 100) was applied using the numpy.convolve function. To exclude artifacts from the moving average at the start and end of the signal, the first and last 50 (½ × window size) data points (∼1.7 h) were removed. The resulting smoothed signal was used for further analysis and graphical representation. The data analysis steps are shown in Fig. 1B. The scipy.signal.find_peaks function was used to detect the oscillation peak timepoints. The scripts are publicly available at https://github.com/flo-sti/cyano-backscatter-oscillation-analysis.

### Data analysis and visualization

The temperature coefficient *Q*_10_ values for the evaluation of temperature compensation for frequency were calculated using the following formula: $Q_{10}={\left(\frac{R_{2}}{R_{1}}\right)}^{\left(\frac{10^{\circ }C}{T_{2}-T_{1}}\right)},$ where the frequency ($R_{x}$) is determined by calculating the period measured in the experiment and $T_{x}$ is the corresponding temperature at which the experiment was conducted.

### Data processing

#### Wavelet transform

Wavelet transformation was performed using Python. The script can be found at https://github.com/flo-sti/cyano-backscatter-oscillation-analysis.

Suppose $x_{n}$ is a discrete time series of *N* observations {$\left\{x_{n,}n=0,\;\ldots ,\;N-1\right\}$, n=0,…,N−1} with a uniform time step δt. The continuous wavelet transform of the discrete time series $x_{n}$ is defined as


$$W_{n}(s)\;=\overset{N-1}{\underset{n^{\prime }\;=\;0}{\sum }}\;x_{n^{\prime }\;}\;\psi^{*}\left((n^{\prime }\;-\;n)\frac{\;\delta t}{s}\right)\;,$$

where *ψ* is the “wavelet function” and *s* is the wavelet scale. Larger scales stretch the wavelet function, making it sensitive to lower frequencies in the signal. The wavelet power spectrum is defined as the square of the wavelet transform amplitude, $|W_{n}(s)){|}^{2}$.

#### Significance level

Following Torrence and Compo (83), the statistical significance of wavelet power can be assessed against a background power spectrum. A suitable background spectrum is either white noise or red noise. Red noise can be modeled as a univariate lag-1 autoregressive process: $x_{n\;}=\alpha \;x_{n-1}+z_{n},$

where *α* is the assumed lag-1 autocorrelation, and $z_{n}$ is the (Gaussian) white noise.

The wavelet power spectrum of red noise is (83):


$$P_{k}=\;\frac{1\;-\;\alpha^{2}}{1+\alpha^{2}\;-\;2\;\alpha \;\cos\;(2\pi k/N)},$$

where *N* is the number of data points, and *k* is the frequency index. The mean background power spectrum significant at the 5% level is $\frac{1}{2}\;P_{k}\;\chi_{2}^{2}$, where $\chi_{2}^{2}$ is a chi-square distribution with two degrees of freedom.

#### Period determination by Fourier transformation

To determine the period of *Synechocystis* and *Synechococcus* WTs under different temperatures, we employed *scipy.fft* functions, *rfft* and *rfftfreq*. The rfft function was applied to the smoothed oscillation signal (Fig. 3A and B) with zero padding of 4× the number of measurements (*N*) (84). For the rfftfreq function, *N* and the time difference between measurements in hours (*T*; 120 s/3,600 s h^−1^) were given as parameters. The scipy.signal.find_peaks function was used to determine the frequency of the highest (or most likely the peak for circadian oscillation). The period was calculated using the reciprocal of the frequency.

#### Comparison between KaiC3 and backscatter oscillation

To estimate a peak of the P-KaiC3 to total KaiC3 ratio, the arithmetic mean of two technical replicates was calculated at each time point. To approximate oscillations insensitive to outliers, a 5th degree polynomial was fitted to the mean values using the numpy.polyfit function. To compare with the backscatter oscillation, the backscatter was analyzed as previously described, and the oscillation peaks were determined using the scipy.stats.find_peaks function.

## Data Availability

All study data are included in the article and/or the SI Appendix. The analysis code is publicly available at GitHub (https://github.com/rahilgholami/Circadian-rhythmicity and https://github.com/flo-sti/cyano-backscatter-oscillation-analysis).

## Preprints

A preprint of a previous manuscript version was included in the dissertation of LCB (https://docserv.uni-duesseldorf.de/servlets/DerivateServlet/Derivate-69081/231009_Cyanobacterial%20Gene%20Expression_LCB.pdf)
